# Combined Effects of 19 Common Variations on Type 2 Diabetes in Chinese: Results from Two Community-Based Studies

**DOI:** 10.1371/journal.pone.0014022

**Published:** 2010-11-17

**Authors:** Min Xu, Yufang Bi, Yu Xu, Bing Yu, Yun Huang, Lina Gu, Yaohua Wu, Xiaolin Zhu, Mian Li, Tiange Wang, Aiyun Song, Jianing Hou, Xiaoying Li, Guang Ning

**Affiliations:** 1 State Key Laboratory of Medical Genomics, Ruijin Hospital, Shanghai Jiao Tong University School of Medicine, Shanghai, China; 2 Department of Endocrine and Metabolic Diseases, Shanghai Clinical Center for Endocrine and Metabolic Diseases, Ruijin Hospital, Shanghai Jiao Tong University School of Medicine, Shanghai, China; 3 Department of Molecular & Clinical Genetics, Royal Prince Alfred Hospital and Central Clinical School, The University of Sydney, Sydney, Australia; 4 Laboratory of Endocrinology and Metabolism, Institute of Health Sciences, Shanghai Institutes for Biological Sciences, Chinese Academy of Sciences, Shanghai Jiao Tong University School of Medicine, Shanghai, China; National Institute of Child Health and Human Development/National Institutes of Health, United States of America

## Abstract

**Background:**

Many susceptible loci for type 2 diabetes mellitus (T2DM) have recently been identified from Caucasians through genome wide association studies (GWAS). We aimed to determine the association of 11 known loci with T2DM and impaired glucose regulation (IGR), individually and in combination, in Chinese.

**Methods/Principal Findings:**

Subjects were enrolled in: (1) a case-control study including 1825 subjects with T2DM, 1487 with IGR and 2200 with normal glucose regulation; and (2) a prospective cohort with 734 non-diabetic subjects at baseline. The latter was followed up for 3.5 years, in which 67 subjects developed T2DM. Nineteen single nucleotide polymorphisms (SNPs) were selected to replicate in both studies. We found that *CDKAL1* (rs7756992), *SLC30A8* (rs13266634, rs2466293), *CDKN2A/2B* (rs10811661) and *KCNQ1* (rs2237892) were associated with T2DM with odds ratio from 1.21 to 1.35. In the prospective study, the fourth quartile of risk scores based on the combined effects of the risk alleles had 3.05 folds (95% CI, 1.31–7.12) higher risk for incident T2DM as compared with the first quartile, after adjustment for age, gender, body mass index and diabetes family history. This combined effect was confirmed in the case-control study after the same adjustments. The addition of the risk scores to the model of clinical risk factors modestly improved discrimination for T2DM by 1.6% in the case-control study and 2.9% in the prospective study.

**Conclusions/Significance:**

Our study provided further evidence for these GWAS derived SNPs as the genetic susceptible loci for T2DM in Chinese and extended this association to IGR.

## Introduction

Type 2 diabetes mellitus (T2DM) is one of the fastest growing diseases with a major impact on morbidity and premature mortality worldwide. A rapid increase in the prevalence of T2DM and impaired glucose regulation (IGR) has also been observed in China in recent decades [1]. T2DM is a complex disorder characterized by impaired insulin sensitivity and pancreatic β cell dysfunction; and is involved in complicated interactions between genetic variants and environmental factors. Multiple genes have been found involving in the pathogenesis of T2DM. Recently, several genome wide association studies (GWAS) and replicated studies on the common genetic variants in T2DM have been reported in several large white populations [2]–[5] since the first GWAS [6] published. Several new candidate genes (*TCF7L2*, *SLC30A8*, *HHEX*, *CDKAL1*, *CDKN2A/2B*, *IGF2BP2*, *KCNQ1*, *etc.*) have been identified in relation to an increased risk for T2DM. Some studies have implicated that the genetic polymorphisms may be involved in the process of insulin production and/or secretion [4], [7], [8].

IGR includes impaired fasting glucose (IFG) and/or impaired glucose tolerance (IGT). IGR is also known as intermediate hyperglycemia or pre-diabetes and characterized by high blood glucose concentrations, insulin resistance and impaired insulin secretion. Previous studies have shown that 5−10% IGT subjects developed diabetes each year, although, some of them could revert spontaneously to normal glucose tolerance [9], [10]. It would be worthwhile to determine whether the common genetic variations play any role in the pathogenesis of IGR and whether IGR shared the same risk genetic background with T2DM [11], [12].

Despite a moderate effect of individual genetic factors on T2DM and a premature testing for inherited susceptibility based on common risk alleles, the genetic assessment for persons at high risk for T2DM has received much consideration [13]. It is important to understand whether a combination of the major genetic factors would contribute more to T2DM or may be used to stratify high-risk populations [14]–[18].

Given the differences in genetic background (ethnics, geographic ancestries, linkage disequilibrium pattern and risk allele frequencies) [19] and risk factor profiles (body composition and insulin secretion/resistance patterns), it is necessary to replicate the genetic association study in Chinese population to clarify the roles in those susceptible genes. In the present study, we aimed to verify the associations of 19 single nucleotide polymorphisms (SNPs) in 11 genes (*PPARG*, *IGF2BP2*, *CDKAL1*, *SLC30A8*, *CDKN2A/2B*, *HHEX*, *EXT2*, *KCNJ11*, *KCNQ1*, *MTNR1B and TCF2*) with the risk of T2DM and IGR in Chinese population; and followed by the investigation of the combined effect of these genes on the risk of T2DM in both case-control study and prospective cohort.

## Methods

### Ethics statement

This study was approved by the Institutional Review Board of the Ruijin Hospital, Shanghai Jiao Tong University School of Medicine and was in accordance with the principle of the Helsinki Declaration II. The written informed consent was obtained from each participant.

### Study population

Case-control study: The participants were recruited from an ongoing glucose survey in Baoshan District of Shanghai during 2004 to 2008. The study population, design and protocols of this case-control study have been previously described [20], [21]. In brief, we first invited all registered permanent residents aged 40 or above by poster advertisement and by mail to participate in a screening examination. We then collected information on lifestyle, medical history and the use of medications using a questionnaire, performed anthropometrical measurements and 75-g oral glucose tolerance tests (OGTT), and blood and urine sampling. Eventually, we enrolled 5012 subjects who have finished OGTT in the genetic study, which included 2200 subjects with normal glucose regulation (NGR, 844 males and 1356 females), 1478 subjects with IGR (595 males and 892 females) and 1825 T2DM patients (802 males, 1023 females).

Prospective study: Nine hundred and forty-four non-diabetic individuals determined at baseline in 2005 in the Baoshan District were invited to participate in the follow-up examination in 2008. After excluding the subjects with neither DNA samples (n = 190) nor information of glucose metabolism status (n = 20) available, the remaining 734 subjects were selected for the genetic analysis.

### Clinical examination and biochemical analysis

Individual height, weight, and waist and hip circumferences were measured by the experienced physicians. Blood pressure was measured at non-dominant arm in a seated position after a ten-min rest using an automated electronic device (OMRON Model1 Plus, Japan). Three measurements were taken in one min apart and an average of the three was used in analysis. The fasting and 2-h OGTT plasma glucose, serum triglycerides, total cholesterol, high-density lipoprotein and low-density lipoprotein cholesterol were determined using an automated biochemical instrument (Beckman CX-7 Biochemical Autoanalyser, Brea, CA, USA). Fasting serum insulin was measured by radioimmunoassay (Sangon Company, Shanghai, China).

### Definitions

IGR was defined as IFG (Fasting plasma glucose ≥5.6 mmol/l and <7.0 mmol/l) and/or IGT (2-h OGTT plasma glucose ≥7.8 and <11.1 mmol/l). T2DM was diagnosed at fasting plasma glucose ≥7.0 mmol/l and/or 2-h OGTT plasma glucose level ≥11.1 mmol/l and/or treatment with antidiabetic medication (oral agents or insulin injection). A fasting plasma glucose level less than 5.6 mmol/l and a 2-h OGTT plasma glucose level less than 7.8 mmol/l were defined as NGR. The insulin resistance index of the homeostasis model assessment (HOMA-IR) was calculated as fasting plasma insulin (in milliunits per milliliter) × fasting plasma glucose (in millimoles per liter)/22.5 and β-cell function (HOMA-β) was assessed as fasting plasma insulin (in milliunits per milliliter) ×20/(fasting plasma glucose - 3.5) (in millimoles per liter).

### Candidate loci and genotyping

We selected 17 common SNPs from 9 loci that had a nominal to strong association with T2DM in recently published GWAS including: *IGF2BP2* (rs1470579 and rs4402960), *CDKAL1* (rs7756992), *SLC30A8* (rs13266634 and rs2466293), *CDKN2A/2B* (rs564398 and rs10811661), *HHEX* (rs7923837, rs1111875 and rs5015480), *EXT2* (rs1113132, rs11037909 and rs3740878), *KCNQ1* (rs2237892), *MTNR1B* (rs10830963 and rs1387153) and *TCF2* (rs7501939) [2]–[6], [22]–[25]. We also included other 2 loci, *PPARG* (rs1801282) and *KCNJ11* (rs5215) in genotyping, which had been validated as candidate genes for T2DM. We did not include the loci of *JAZF1*, *CDC123-CAMK1D*, *TSPAN8-LGR5*, *THADA*, *ADAMTS9* and *NOTCH2*, which were reported from a meta-analysis [26]. *TCF7L2* (rs12255372, rs7901695, rs7903146 or rs11196205), and *WFS1* (rs6446482, rs10010131) loci were excluded since their minor allele frequencies are less than 5% in Han Chinese according to the HapMap CHB group (http://snp.cshl.org/cgi-perl/gbrowse/hapmap22_B36/). Exclusion also contains the *FTO* loci (rs8050136, rs9939609, rs9930506) since no association of this locus with T2DM has been demonstrated in Chinese [27].

Genomic DNA was extracted from peripheral blood leukocytes with standard phenol/chloroform-based method. All the selected SNPs were genotyped by SNaPshot® Multiplex System (Applied Systems) following the manufacture's protocol. In our study, the call rate was ranged from 94% (rs2466293) to 99% (rs3740878) in the case-control study, and from 97% (rs1801282) to 99% (rs564398) in the prospective cohort. There is no significant difference of SNP calling between the case and the control groups. The average consensus rate in the duplicate samples (n = 256) was 99.7%, and all the SNPs were in accordance with Hardy-Weinberg equilibrium (all *P*≥0.01, Table S1).

### Risk score

The risk score was calculated on the basis of SNPs that were significantly associated with T2DM in the present case-control study. We assumed the additive genetic model [28] for each SNP, applying a linear weighing of 0, 1, and 2 to genotypes containing 0, 1, or 2 risk alleles, respectively. Three logistic regression models with different adjustments were used to investigate effect of risk scores on T2DM and IGR in the case-control analysis and on incident diabetes in the prospective analysis, respectively. Multiplicative interactions between conventional risk factors and the risk scores were tested using the likelihood ratio test. To measure the discriminative improvement attributable to the risk score, we plotted receiver-operating characteristic curves (ROCs) for a logistic regression model including conventional risk factors and a model including conventional risk factors and the genetic risk score [29]. The conventional model included age (continuous), gender, family history of diabetes (yes or no) and BMI (continuous).

### Statistical analysis

Deviation from Hardy-Weinberg equilibrium for genotypes at individual locus was assessed using the Chi-square test. A multiple logistic regression model was used to investigate the individual effect of these genes on IGR and T2DM. These analyses were based on additive, recessive and dominant models, and adjusted for age, gender and BMI. The statistical analyses were performed using SAS version 8.1 (SAS Institute, Cary, NC). In order to avoid any potential spurious result in our association replications, the most conservative Bonferroni correction was used to ensure a high stringent condition for any positive result. *P*<0.0026 (0.05 divided by 19, the total number of SNPs studied) was considered significant. LD estimation of the SNPs was obtained using Haploview version 3.32 (http://www.broad.mit.edu/mpg/haploview/). Current sample size, minor allele frequencies observed in the present study and the previously reported odds ratios (ORs) for T2DM was used for statistical power estimation (Table S1).

## Results

### The clinical characteristics of the study subjects

The case-control study had a total of 5512 subjects, including 2200 subjects (39.9%) with NGR, 1487 (27.0%) with IGR and 1825 (33.1%) T2DM patients. The characteristics of the participants were shown in Table 1.

**Table 1:** Characteristics of the participants in the case-control study.

	The case-control sample set (n = 5512)			
	NGR	IGR	T2DM	*P* *
Male/Female, n (M, %)	844/1356 (38.4)	595/892 (40.0)	802/1023 (43.9)	<0.0001
Age (years)	59.3±9.6	61.0±9.4	63.3±9.7	<0.0001
Body mass index (kg/m^2^)	24.3±3.3	25.5±3.4	26.3±3.8	<0.0001
Systolic blood pressure (mmHg)	131±20	140±22	147±22	<0.0001
Diastolic blood pressure (mmHg)	77±10	80±10	81±11	<0.0001
Fasting plasma glucose (mmol/l)	4.9±0.5	5.5±0.6	7.7±2.5	<0.0001
OGTT-2h plasma glucose (mmol/l)	6.0±1.1	8.3±1.5	16.1±5.2	<0.0001
Fasting serum insulin (µU/ml)	4.3 (4.0–4.7)	5.0 (4.6–5.4)	6.4 (5.9–6.9)	<0.0001
HOMA_IR (%)	1.17 (0.73–1.76)	1.74 (1.03–2.69)	2.88 (1.72–4.84)	<0.0001
HOMA_β (%)	82.1(51.7–124.9)	74.4(43.1–123.5)	50.2(25.3–90.6)	<0.0001
Current smoking, yes, n (%)	465 (21.1)	276 (18.6)	385 (22.7)	0.01
Current alcohol intake, yes, n (%)	353 (16.9)	239 (16.2)	299 (18.4)	0.06
Diabetes family history, yes, n (%)	258 (12.3)	226 (15.4)	430 (25.3)	<0.0001

Data are means ± SD or median (interquartile) or number (percentage).

*Based on ANOVA for continuous variables and χ^2^ for categorical variables. HOMA_IR, homeostasis model assessment of insulin resistance; and HOMA_β, homeostasis model assessment of β-cell function.

In the prospective study, of the 734 non-diabetic subjects at baseline, 67 subjects turned to T2DM in 3.5 years. The clinical characteristics of the prospective study subjects were shown in Table S2.

### Individual effects of polymorphisms on IGR and T2DM

#### The case-control study

The characteristics of the 19 risk loci and their associations with IGR and T2DM were shown in Table 2. Three heredity models (additive, recessive or dominant) were introduced to study the associations between the SNPs and IGR or T2DM. SNPs rs10811661 (*CDKN2A/2B*) and rs2466293 (*SLC30A8*) were associated with increased risk in both IGR and T2DM. SNP rs7756992 (*CDKAL1*) was associated with T2DM, but not IGR. SNPs rs13266634 (*SLC30A8*) and rs2237892 (*KCNQ1*) were nominally associated with IGR and statistically significantly associated with T2DM (*P*<00001); whereas, two SNPs rs1470579 and rs4402960 (*IGF2BP2*), and two SNPs rs5215 (*KCNJ11*) and rs7501939 (*TCF2*) were nominally associated with T2DM. SNPs rs1111875 (*HHEX*) and rs10830963 (*MTNR1B*) were only associated with the risk of IGR, not T2DM. All the analysis was based on the adjustment for age, gender and BMI.

**Table 2:** Gene Loci, minor allele frequency and associations of the 19 SNPs with impaired glucose metabolism in the case and control subjects.

SNP	Gene	Major/Minor Allele	Minor allele frequency			IGR vs. NGR				T2DM vs. NGR			
			NGR	IGR	T2DM	OR_add_ (95% CI)	*P*_add_ †	*P*_rec_ †	*P*_dom_ †	OR_add_(95% CI)	*P*_add_ †	*P*_rec_ †	*P*_dom_ †
			n = 2200	n = 1487	n = 1825								
rs1801282	*PPARG*	C/G^a^	0.079	0.064	0.071	0.75 (0.61–0.93)	**0.007**	**0.01**	**0.02**	0.87 (0.72–1.07)	0.18	0.13	0.30
rs1470579	*IGF2BP2*	A/C a	0.243	0.251	0.267	1.06 (0.94–1.19)	0.37	**0.01**	0.67	1.13 (1.01–1.27)	**0.03**	0.71	0.08
rs4402960	*IGF2BP2*	G/T a	0.232	0.244	0.257	1.08 (0.96–1.22)	0.20	0.28	0.26	1.15 (1.03–1.29)	**0.01**	**0.05**	**0.03**
**rs7756992**	***CDKAL1***	G a/A	0.492	0.503	0.536	1.05 (0.95–1.16)	0.35	0.52	0.33	**1.21 (1.10–1.34)**	**0.0001** ‡	**0.003**	**0.0004** ‡
**rs13266634**	***SLC30A8***	C^a^/T	0.468	0.439	0.430	1.11 (1.01–1.22)	**0.03**	**0.04**	0.13	**1.20 (1.09–1.32)**	**0.0002** ‡	**0.002** ‡	**0.002** ‡
**rs2466293**	***SLC30A8***	A/G^a^	0.359	0.396	0.404	**1.17 (1.06**–**1.30)**	**0.0025** ‡	**0.0016** ‡	0.49	**1.25 (1.13–1.38)**	**<0.0001** ‡	**<0.0001** ‡	**0.01**
**rs10811661**	***CDKN2A/2B***	C/T a	0.498	0.543	0.568	**1.23 (1.11–1.36)**	**<0.0001** ‡	**0.0006** ‡	**0.01**	**1.35 (1.22–1.49)**	**<0.0001** ‡	**<0.0001** ‡	**<0.0001** ‡
rs564398	*CDKN2A/2B*	T/C^a^	0.412	0.507	0.509	1.02 (0.87–1.19)	0.79	0.37	0.70	1.05 (0.90–1.22)	0.52	0.26	0.60
**rs1111875**	***HHEX***	A/G a	0.280	0.313	0.292	**1.21 (1.08–1.36)**	**0.0007** ‡	**0.0005** ‡	0.09	1.10 (0.99–1.23)	0.08	0.14	0.41
rs7923837	*HHEX*	A/G^a^	0.209	0.222	0.223	1.09 (0.96–1.23)	0.17	0.06	0.66	1.08 (0.96–1.22)	0.21	0.12	0.89
rs5015480	*HHEX*	T/C^a^	0.164	0.177	0.173	1.12 (0.98–1.28)	0.11	0.07	0.47	1.07 (0.94–1.22)	0.30	0.24	0.27
rs1113132	*EXT2*	C^a^/G	0.427	0.404	0.402	1.09 (0.98–1.21)	0.10	**0.01**	0.40	1.07 (0.97–1.18)	0.21	0.13	0.21
rs11037909	*EXT2*	T^a^/C	0.411	0.414	0.416	1.11 (1.00–1.23)	0.06	**0.02**	0.46	1.08 (0.98–1.19)	0.14	0.33	0.15
rs3740878	*EXT2*	A^a^/G	0.447	0.443	0.429	1.05 (0.95–1.16)	0.38	0.14	0.77	1.06 (0.97–1.17)	0.21	0.22	0.42
rs5215	*KCNJ11*	T/C a	0.393	0.380	0.411	0.97 (0.88–1.07)	0.55	0.87	0.10	1.12 (1.01–1.23)	**0.03**	**0.04**	0.38
**rs2237892**	***KCNQ1***	a C/T	0.359	0.335	0.302	1.17 (1.05–1.30)	**0.005**	0.15	**0.01**	**1.35 (1.21–1.50)**	**<0.0001** ‡	**0.001** ‡	**<0.0001** ‡
rs10830963	*MTNR1B*	C/G a	0.421	0.451	0.430	1.15 (1.04–1.28)	**0.007**	0.06	**0.006**	1.03 (0.93–1.14)	0.64	0.21	0.79
rs1387153	*MTNR1B*	C/T^a^	0.413	0.436	0.425	1.10 (0.99–1.21)	0.08	0.13	0.06	1.03 (0.93–1.13)	0.63	0.25	0.75
rs7501939	*TCF2*	C/T a	0.251	0.265	0.272	1.06 (0.95–1.19)	0.32	0.29	0.10	1.15 (1.03–1.29)	**0.01**	**0.02**	**0.03**

The SNPs were shown with the risk allele and minor allele frequencies (MAF) in normal glucose regulation (NGR), impaired glucose regulation (IGR) and type 2 (T2DM). The odds ratios of the risk allele (OR_add_) were calculated using an additive genetic model that in logistic regression was multiplicative on the OR scale. *P* values were calculated using the additive, recessive (rec) or dominant (dom) genetic model, respectively.

† , adjustment including age, gender and BMI.

a , Risk allele in previously reported studies. The underlined allele is the risk allele supported by the present study.

‡ , indicating *P* value remains statistical significant after Bofferoni correction (*P* = 0.05/19 = 0.0026).

#### The prospective study

The genotype frequencies and individual risk for incident diabetes were shown in Table 3. The risk allele of SNPs rs10811661 (*CDKN2A/2B*), rs13266634 (*SLC30A8*) and rs2466293 (*SLC30A8*) increased the risk of incident T2DM by 94%, 88% and 152%, respectively, in the recessive model after adjustment for the effect of age, gender and BMI. The risk allele C of rs1387153 (*MTNR1B*) was associated with the increased risk of T2DM by 85% in the dominant model.

**Table 3:** Genotype frequencies of 19 SNPs and individual risks for incident T2DM among the participants who were nondiabetic at baseline.

SNP	Region	Major/Minor Allele	Genotype frequencies						*OR_add_*	*P_add_*	*OR_rec_*	*P_rec_*	*OR*_domi_	*P_dom_*
			Non-Diabetes			Incident Diabetes								
			MM	Mm	mm	MM	Mm	mm						
rs1801282	*PPARG*	C/G^a^	3 (0.5)	70(10.8)	576(88.8)	0(0)	5(7.8)	59(92.2)	0.69(0.28–1.71)	0.42	0.001 (0. 001–999.999)	0.99	0.71(0.27–1.83)	0.47
rs1470579	*IGF2BP2*	A/C^a^	37(5.6)	238(36.3)	381(58.1)	2(3.0)	25(37.3)	40(59.7)	0.91(0.58–1.41)	0.66	0.57(0.13–2.59)	0.47	0.96(0.57–1.61)	0.87
rs4402960	*IGF2BP2*	G/T^a^	34(5.2)	230(35.2)	390(59.6)	3(4.5)	22(32.8)	42(62.7)	0.92(0.59–1.43)	0.71	0.91(0.27–3.08)	0.87	0.91(0.53–1.53)	0.71
rs7756992	*CDKAL1*	A/G^a^	154(23.5)	338(51.5)	164(25.0)	16(23.9)	30(44.8)	21(31.3)	0.93(0.65–1.35)	0.71	1.08(0.60–1.97)	0.79	0.79(0.45–1.38)	0.40
**rs13266634**	***SLC30A8***	C/T^a^	173(26.6)	359(55.1)	119(18.3)	29(43.5)	26(40.0)	11(16.5)	1.38(0.91–2.09)	0.13	**1.88(1.08–3.28)**	**0.02**	1.00(0.48–2.09)	0.99
**rs2466293**	***SLC30A8***	A/G^a^	72(11.0)	329(50.6)	250(38.4)	15(23.8)	28(42.9)	22(33.3)	1.43(0.99–2.27)	0.07	**2.52(1.29–5.06)**	**0.009**	1.23(0.70–2.18)	0.47
**rs10811661**	***CDKN2A/2B***	C/T^a^	172(26.0)	334(50.5)	156(23.5)	26(38.8)	27(40.3)	14(20.9)	**1.46(1.01–2.11)**	**0.047**	**1.94(1.14–3.31)**	**0.01**	1.27(0.67–2.41)	0.46
rs564398	*CDKN2A/2B*	T/C^a^	7(1.1)	128(19.3)	528(79.6)	2(3.0)	13(19.4)	52(77.6)	1.18(0.69–2.02)	0.56	2.37(0.46–12.27)	0.30	1.11(0.60–2.05)	0.74
rs1111875	*HHEX*	A/G^a^	51(7.7)	257(38.9)	352(53.3)	6(9.0)	32(47.8)	29(43.3)	1.36(0.93–2.01)	0.12	1.23(0.50–3.02)	0.66	1.58(0.94–2.66)	0.08
rs7923837	*HHEX*	A/G^a^	24(3.7)	233(35.6)	397(60.7)	3(4.6)	21(32.3)	41(60.8)	1.03(0.65–1.62)	0.91	1.26(0.37–4.32)	0.72	1.00(0.58–1.71)	0.99
rs5015480	*HHEX*	T/C^a^	23(3.5)	183(27.6)	456(68.9)	4(6.0)	22(32.8)	41(61.2)	0.95(0.71–1.25)	0.69	0.93(0.41–2.11)	0.87	0.94(0.67–1.30)	0.69
rs1113132	*EXT2*	C^a^/G	212(32.2)	330(50.2)	116(17.6)	29(43.3)	30(44.8)	8(11.9)	1.47(1.00–2.15)	0.05	1.66(0.99–2.79)	0.06	1.58(0.73–3.44)	0.25
rs11037909	*EXT2*	T^a^/C	210(32.1)	334(51.0)	111(16.9)	27(40.9)	32(48.5)	7(10.6)	1.43(0.97–2.12)	0.07	1.52(0.90–2.58)	0.12	1.73(0.76–3.94)	0.19
rs3740878	*EXT2*	A^a^/G	211(32.2)	330(50.3)	115(17.5)	26(39.4)	32(48.5)	8(12.1)	1.33(0.91–1.96)	0.14	1.42(0.83–2.40)	0.20	1.54(0.71–3.33)	0.28
rs5215	*KCNJ11*	T/C^a^	103(15.8)	253(36.4)	298(45.6)	10(15.2)	24(36.4)	32(48.5)	0.94(0.65–1.35)	0.73	1.01(0.50–2.07)	0.97	1.17(0.68–2.00)	0.58
rs2237892	*KCNQ1*	aC/T	272(41.7)	300(46.0)	80(12.3)	30(45.5)	33(50.0)	3(4.6)	1.35(0.90–2.02)	0.14	1.19(0.71–2.01)	0.51	3.21(0.98–10.53)	0.05
rs10830963	*MTNR1B*	C/G^a^	118(18.0)	337(31.5)	200(30.5)	10(15.2)	31(47.0)	25(37.9)	0.80(0.54–1.18)	0.26	0.91(0.45–1.86)	0.80	0.68(0.40–1.15)	0.15
rs1387153	*MTNR1B*	C/T^a^	119(18.2)	324(49.5)	212(32.4)	8(12.1)	28(42.4)	30(45.5)	**1.52 (1.02–2.27)**	**0.04**	1.39(0.64–3.03)	0.41	**1.85 (1.11–3.13)**	**0.02**
rs7501939	*TCF2*	C/T^a^	39(6.0)	235(36.1)	377(57.9)	7(10.8)	16(24.6)	42(64.6)	0.99()0.65–1.53	0.98	2.32(0.98–5.53)	0.06	0.77(0.45–1.32)	0.34

Data are shown as genotype frequencies or odds ratio, 95% confidence interval.

The logistic regression model was used to obtain the odds ratios of the risk allele for incident diabetes based on additive (add), recessive (rec) or dominant (dom) model, respectively. The *P* values were adjusted for age, gender and BMI.

a Risk allele. MM, the homozygote of the risk alleles.

### Genetic risk score and risk of type 2 diabetes

The risk score was calculated based on SNPs rs7756992 (*CDKAL1*), rs2466293 (*SLC30A8*), rs10811661 (*CDKN2A/2B*) and rs2237892 (*KCNQ1*), which were statistically significantly associated T2DM in the case-control study. The risk score was calculated by summing up the number of risk alleles for each participant who had the genotyping information of these 4 SNPs (534 participants were excluded from calculation because of incomplete genotype information). We included SNP rs2466293 of *SLC30A8* to calculate the risk score since it was reported to be associated with T2DM in Chinese in our previous study [30] and the correlation between SNP rs13266634 and rs2466293 was moderate (r-squared  = 0.49) (Table S3). The risk scores were significantly higher in T2DM and IGR than that in NGR. The mean risk scores for T2DM, IGR and NGR were 4.45, 4.24 and 3.99, respectively (*P*<0.0001) after adjustment for age, gender, BMI, diabetes family history, current smoking and alcohol intake in the case-control analysis. Similarly, the mean risk score was 4.81 for the incident diabetic patients and 4.33 for the non-diabetics in the prospective study, and the difference reached statistical significant (*P* = 0.02), after the adjustment for the same factors as above.

**Figure 1:**
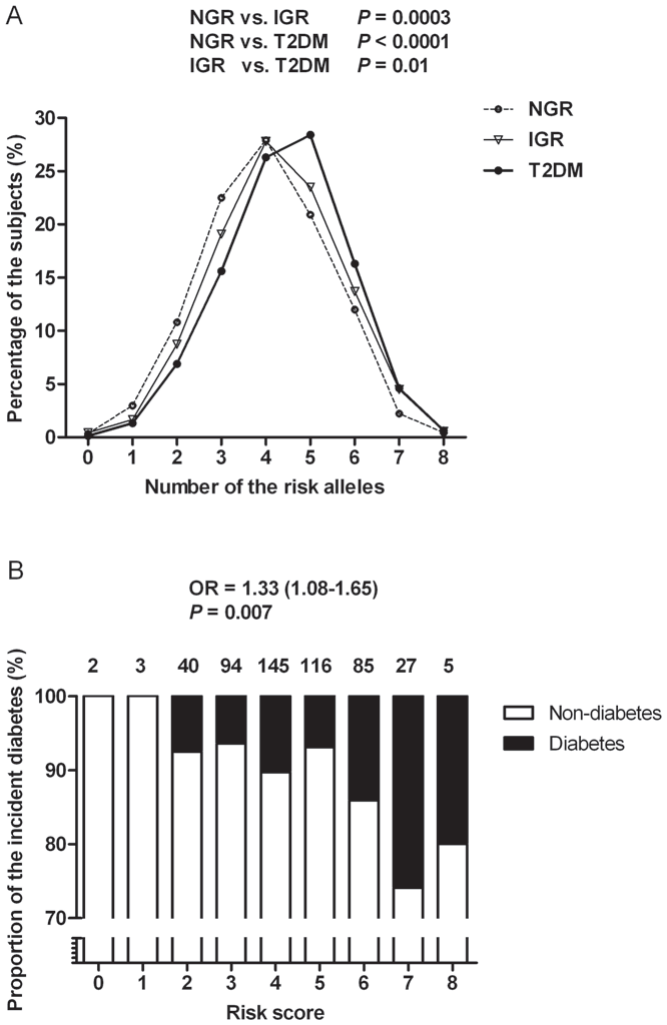
Distribution of the genetic risk score and different glucose metabolism status. Panel A, the case-control study. The percentage of different glucose metabolism status according to the number of risk alleles. *P* values were calculated by Chi-square analysis. Panel B, the prospective study. The percentage of incident T2DM and remaining non-diabetes according to the risk score. The numbers above the bars are the subjects with the corresponding risk score. With per 1 risk allele increasing, the risk for incident T2DM increases by 33% (*P* = 0.007), after adjusted for age, gender, BMI, diabetes family history, current smoking and alcohol intake.

The subjects with T2DM or IGR had more risk alleles than those with NGR (both *P≤*0.0003) (Figure 1A). Also, the T2DM incidence was increased significantly along with the increased number of risk alleles (Figure 1B).

In the case-control study, we performed the logistic regression analysis for the association between the risk scores and T2DM and IGR in both continuous and category patterns (Table 4). Compared with the first quartile of risk scores, the fourth quartile has 1.53 and 2.29 folds higher risk of IGR and T2DM, respectively, after adjustment for age, gender, BMI, current smoking, alcohol intake (Model 2). The category analysis, in which the risk score was classified by quartiles (0–3, 4, 5, 6–8) yielded similar results (Table 4). Furthermore, possible interactions between genetic risk scores and the clinical risk factors in the case-control association study were explored in stratified analysis and by adding interaction terms to logistic regression models (Table S4). We stratified the study subjects by quartiles of BMI (≤22.9, 23.0–25.0, 25.1–27.4, ≥27.5), quartiles of HOMA_β (≤39.1, 39.2–69.4, 69.5–114.6, ≥114.7) and family history (yes or no). The *P* values for interaction were shown in Table S4. In each of the stratification, the increased risk score was associated with the prevalence of T2DM (all *P _for trend_*<0.05, Table S4).

**Table 4:** The risk of impaired glucose regulation and type 2 diabetes in relation to gene risk scores.

			Impaired glucose regulation	*P1 _for trend_*	Type 2 diabetes	*P2 _for trend_*
**Cross-sectional**						
Continuous, per 1 risk score						
Model 1			1.13 (1.08–1.20)	<0.0001	1.26 (1.20–1.33)	<0.0001
Model 2			1.18 (1.11–1.25)	<0.0001	1.28 (1.21–1.35)	<0.0001
Classed by quartiles of the risk score						
Model 1	Q1	0–3	1	0.0002	1	<0.0001
	Q2	4	1.22 (1.01–1.47)		1.44 (1.20–1.73)	
	Q3	5	1.38 (1.13–1.68)		2.08 (1.72–2.50)	
	Q4	6–8	1.57 (1.26–1.95)		2.24 (1.83–2.75)	
Model 2	Q1	0–3	1	0.0003	1	<0.0001
	Q2	4	1.25 (1.03–1.52)		1.51 (1.23–1.84)	
	Q3	5	1.45 (1.18–1.78)		2.23 (1.82–2.74)	
	Q4	6–8	1.53 (1.22–1.92)		2.29 (1.83–2.86)	

Values are odds ratio (95% confidence interval). *P1*_for trend_ value, for the risk of impaired glucose regulation, we defined participants with normal glucose regulation as 0 and impaired glucose regulation as 1, not including type 2 diabetic patients in the analysis; while *P2*_for trend_ value, for the risk of type 2 diabetes, we defined normal glucose regulation as 0 and type 2 diabetes as 1. Q1, quartile 1; Q2, quartile 2; Q3, quartile 3; Q4 quartile 4.

Model 1, unadjusted;

Model 2, adjusted for age, gender BMI, diabetes family history, current smoking and alcohol intake.

In the prospective study, the same models were introduced (Table 5). The fourth quartile of risk scores had 3.05 folds (95% CI, 1.31–7.12) higher risk for incident T2DM as compared with the first quartile, after adjustment for age, gender, BMI, diabetes family history, current smoking and alcohol intake (Model 2, Table 5).

**Table 5:** The predictive effect of the risk score on incident diabetes in the prospective study.

Model 1			Model 2
Continuous, per 1 risk score			
ORs		1.32 (1.07–1.63)	1.33 (1.08–1.68)
*P1 _for trend_*		0.009	0.007
		
Classed by quartiles of the risk score			
	Q1 (0–3)	1	1
	Q2 (4)	1.67 (0.67–3.94)	1.60 (0.66–3.88)
	Q3 (5)	1.07 (0.40–2.87)	1.20 (0.44–3.27)
	Q4 (6–8)	2.98 (1.30–6.83)	3.05 (1.31–7.12)
*P2 _for trend_*		0.02	0.03

Values are odds ratio (95% confidence interval). *P _for trend_* values, for the risk of incident type 2 diabetes, we defined subjects remaining non-diabetic as 0 and the incident type 2 diabetes as 1. Q1, quartile 1; Q2, quartile 2; Q3, quartile 3; Q4 quartile 4.

Model 1, unadjusted;

Model 2, adjusted for age, gender BMI, diabetes family history, current smoking and alcohol intake.

### Discriminative improvement attributable to the risk score

**Figure 2:**
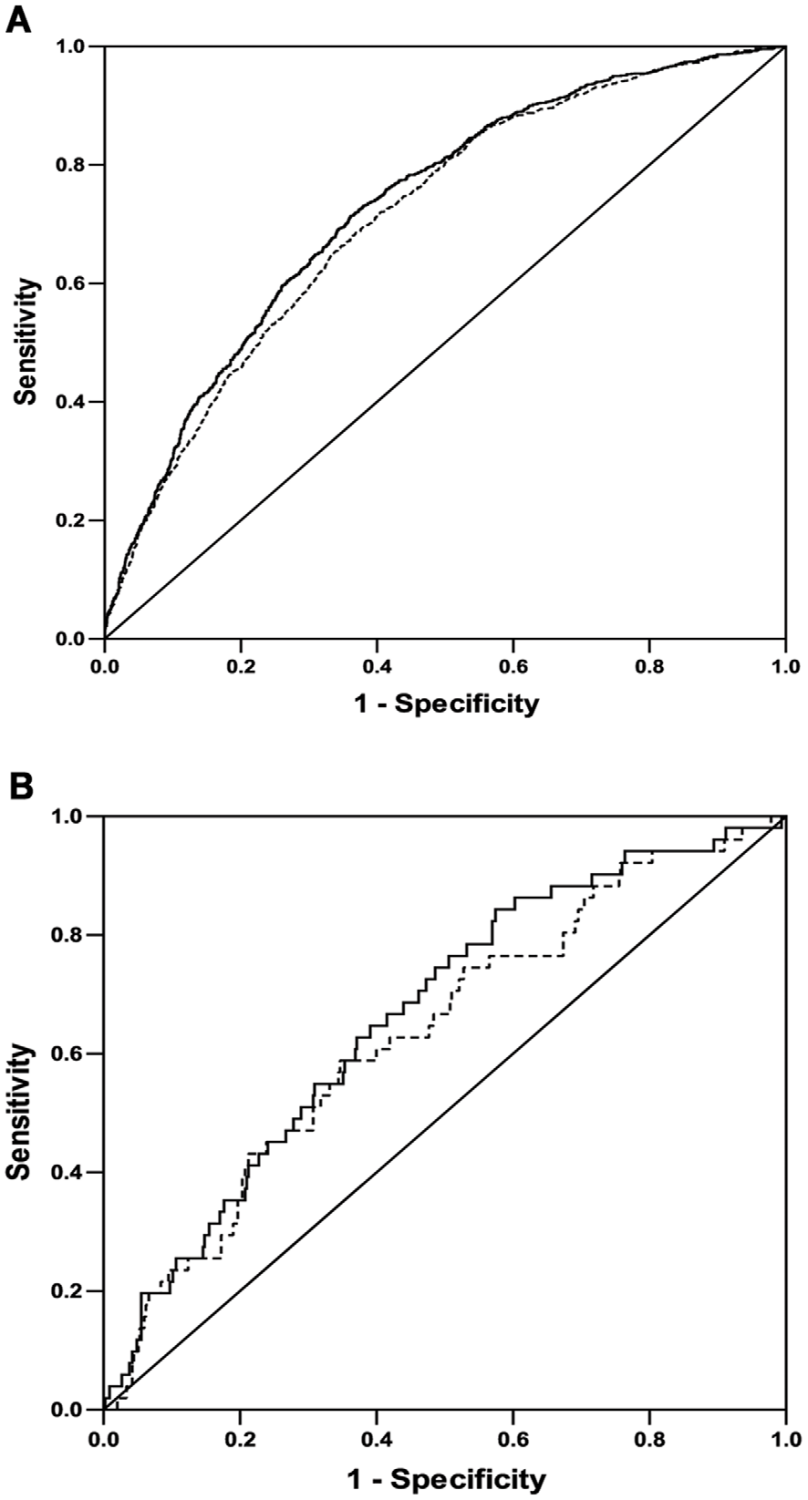
Discriminative improvements attributable to the risk score. Panel A, the case-control study. The combined effect of genetic risk score and clinical factors on T2DM increased by 1.6% compared to clinical factors. The area under ROC was 0.714 for clinical factors and 0.730 for combined risk score and clinical factors (both *P*<0.0001). Panel B, the prospective study. The discriminative improvement for incident T2DM by combining the genetic risk score was 2.9% compared with clinical factors (the area under ROC was 0.634 for clinical factors and 0.663 for combined risk factors, *P* = 0.002 and <0.0001, respectively). The black line represented the combined effect of clinical factors and risk score, and the dotted line was the effect of clinical factors. The clinical factors included age (continuous), gender, family history of diabetes (yes or no) and BMI (continuous). The risk score was categorized as quartiles.

The combined effect of genetic and clinical risk factors on T2DM was shown in Figure. 2. The area under ROC was 0.714 for clinical risk factors alone and 0.730 for combined genetic risk score and clinical risk factors (both *P*<0.0001). Thus, the combined effect was only increased by 1.6% as compared with clinical factors in the case-control study (Figure 2A). In the prospective study, the discriminative improvement for incidence of T2DM by combining the genetic risk score was increased by 2.9% as compared with clinical factors alone (Figure 2B, the area under ROC was 0.634 for clinical factors and 0.663 for combined risk factors, *P* = 0.002 and <0.0001, respectively).

## Discussion

In our present study, the findings support the individual associations of *CDKN2A/2B* (rs10811661), *SLC30A8* (rs13266634 and rs2466293), *CDKAL1* (rs7756992) and *KCNQ1* (rs2237892) with not only T2DM but also IGR in a case-control study. We also confirmed the predictive effect of *CDKN2A/2B* (rs10811661), *SLC30A8* (rs13266634 and rs2466293) on the incident T2DM in the 3.5 year follow-up study. Furthermore, we found that the combination of the risk alleles demonstrated a more robust association with T2DM and IGR than a single one after adjustment for the common clinical risk factors, such as age, gender, BMI and diabetes family history in both case-control and prospective studies. The combined genetic risk scores only had a discriminative improvement of 2.9% for incidence of T2DM as compared with clinical risk factors alone.

We observed a significant association between T2DM and *SLC30A8* (rs13266634 and rs2466293) and *CDKN2A/2B* (rs10811661) in not only the case-control study but also the prospective study. These findings were consistent with what have been observed in a large sample-size Caucasian population in Denmark [31] and several populations in Asia [30], [32]–[34]. Recently, we have verified that *SLC30A8* gene is a susceptible locus for T2DM in Chinese population [30]. We confirmed the rs13266634 was associated with T2DM and reported that rs2466293 (one of the tagger SNPs of *SLC30A8*) was nominally associated with T2DM. Moreover, SNP rs13266634 is correlated with glucose-stimulated insulin secretion. A similar observation is also confirmed in a Japanese population [32]. In a case-control replication study of 6719 Asians including a Chinese cohort from Hong Kong and two Korean cohorts, these candidate genes have critical contribution to T2DM as compared with Caucasians [33]. Functional studies [35], [36] found that zinc transporter 8 (ZnT8) is required for normal insulin crystallization and insulin processing and secretion. The R allele of rs13266634 (W325R) may increase T2DM risks [35]. Further studies which focus on small molecule activators that target ZnT8 may thus represent an interesting means to treat insulin secretary deficiency in T2DM.

SNP rs10811661 is located at 125 kilo-bases upstream of the *CDKN2A/2B* gene. Given the prior knowledge on SNP function, we assumed that SNP rs10811661 might exert its effect on transcription directly or indirectly through an unknown locus which have high LD with this variant. The *CDKN2A/2B* genes are expressed in adipocytes and pancreatic islets [6]. *CDKN2A/2B* encodes for p16INK4a, a tumor suppressor influencing pancreatic β-cell proliferation [37], [38].It is possible for a causal variant situated in *CDKN2A/2B* to increase the susceptibility of T2DM through β-cell mass reduction and subsequent insulin release impairment in the sates with increased insulin demand.

*KCNQ1* gene was believed to be a confirmed risk loci for T2DM in Chinese [39], [40]. In the present study, we confirmed that SNPs rs2237892 in *KCNQ1* was in relation to T2DM and IGR with the odds ratio of 1.35 and 1.17 respectively in the case-control analysis, but not in the prospective study. The predictive effect of *KCNQ1* gene for incident diabetes and the potential mechanism of this gene in the pathogenesis of T2DM remain to be explored.

We found some evidence of combined effect of those risk alleles on T2DM in both case-control and prospective studies. These results are consistent with those reported by Scott et al. [3] and other groups [14]–[18]. In Scott's study, they examined the combined effect of ten risk variants in a GWAS of Europeans, in which they found a fourfold variation in T2DM risk from the lowest to highest predicted risk groups. However, they pointed out that the predictions based on their data might be biased owing to a likely overestimation of ORs because of enrichment for familial T2DM and exclusion of individuals with impaired glucose tolerance or impaired fasting glucose. The risk score in our study improved case–control discrimination beyond what the clinical risk factors could provide, but the magnitude of this improvement was small. This was consistent with other studies performed in prospective populations that provided the joint effect of multiple risk loci and the combined prediction on incident T2DM [14]–[18]. Lyssenko et al. [16] suggested that the addition of genotyping data from the known DNA variants to clinical risk factors, including a family history of diabetes, had a minimal, albeit statistically significant effect on the prediction of future T2DM and the assessment of genetic risk factors is more meaningful in the early life. However, a replication in a larger prospective population would be more convinced to affirm whether combinations of risk alleles from these variants provide a better predictive and diagnostic potential in Chinese.

We included subjects with IGR (impaired fasting glucose and impaired glucose tolerance) in the present study. Few studies were concerned about the association of these GWAS variations with IGR [11]. IFG and/or IGT were predisposed to diabetes; however, whether the IGR and T2DM shared the same spectrum of genetic variations is not well characterized. Here in our study, we found that majority of the SNPs that are associated with T2DM was also conferred the risk of IGR. Our study provided evidence that IGR might have similar background of susceptible genetic variations. However, because the IGR included IFG and IGT which may have different genetic etiology [41], [42], more prospectively-designed association studies with large sample size and more SNPs included are needed in the near future.

Our present study has strength and limitation to be addressed. The main strength of the present study was that we explored the combining effect of those susceptible genes in both a case-control study with a moderate sample size and a 3.5-year follow-up study. We speculated that the joint effect of the genetic variations, which were validated in our study, provided a more strong association with risk of T2DM and IGR. This study extended the knowledge about the genetic factors and the pathogenesis of T2DM beyond the Caucasian population. There are some limitations that should be addressed in this study. The sample size for the prospective study was relatively small and the cases of incident T2DM were limited. Only two of the SNPs that were found to be significantly associated with T2DM in the case-control analysis were validated in the prospective study.

In conclusion, our study affirmed the associations of SNPs in *CDKN2A/2B, SLC30A8, KCNQ1,* and *CDKAL1* genes with the risk of IGR and T2DM in a case-control study; and stronger associations were found when the risk alleles combined. Our study provided the further evidence of that these GWAS derived genetic susceptible variations are also important for T2DM in Chinese and extended the association of these variations with IGR.

## Supporting Information

Table S1 SNPs genotyped in the Chinese Han population
(0.05 MB DOC)

Table S2 The baseline clinical characteristics of the participants in the prospective study.
(0.04 MB DOC)

Table S3 Standardized pair-wise linkage disequilibrium coefficients D' and r2 of the SNPs.
(0.05 MB DOC)

Table S4 The risk of type 2 diabetes in relation to gene risk scores by stratification analysis.
(0.11 MB DOC)
