# Supplementary material for: Combined Effects of 19 Common Variations on Type 2 Diabetes in Chinese: Results from Two Community-Based Studies
Source: PLoS One. 2010 Nov 17;5(11):e14022. doi: 10.1371/journal.pone.0014022 (PMC2984434; doi:10.1371/journal.pone.0014022)
Supplement: Table S1 — SNPs genotyped in the Chinese Han population (0.05 MB DOC) [file pone.0014022.s001.doc]

**Table S1.**

| db SNP ID | Gene | Major/minor  allele | ORs previously reported (Refs) | *P* (HWE) | MAF | | | | | Power* |
| --- | --- | --- | --- | --- | --- | --- | --- | --- | --- | --- |
|  |  |  |  |  | Current Pooled Sample | CHB | JPT | CEU | YRI |  |
| rs1470579 | *IGF2BP2* | A/C^a^ | 1.17 (5) | 0.37 | C: 0.249 | C: 0.256 | C: 0.352 | C: 0.292 | A: 0.133 | 0.90 |
| rs4402960 | *IGF2BP2* | G/T^a^ | 1.14 (6) | 0.49 | T: 0.234 | T: 0.222 | T: 0.311 | T: 0.292 | G: 0.450 | 0.88 |
| rs7756992 | *CDKAL1* | G^a^/A | 1.25 (4) | 0.30 | A: 0.497 | G: 0.467 | G: 0.466 | G: 0.250 | A: 0.367 | 0.99 |
| rs10811661 | *CDKN2A/2B* | T^a^/C | 1.20 (6) | 0.71 | C: 0.498 | C: 0.405 | C: 0.433 | C: 0.208 | C: 0.000 | 0.99 |
| rs564398 | *CDKN2A/2B* | T/C^a^ | 1.12 (6) | 0.15 | C: 0.119 | C: 0.067 | C: 0.102 | C: 0.375 | C: 0.000 | 0.39 |
| rs1111875 | *HHEX* | A/G^a^ | 1.19 (2) | 0.75 | G: 0.281 | G: 0.322 | G: 0.409 | G: 0.442 | G: 0.142 | 0.97 |
| rs5015480 | *HHEX* | T/C^a^ | 1.13 (6) | 0.55 | C: 0.168 | C: 0.211 | C: 0.189 | T: 0.448 | T: 0.432 | 0.56 |
| rs7923837 | *HHEX* | A/G^a^ | 1.22 (2) | 0.24 | G: 0.208 | G: 0.211 | G: 0.193 | A: 0.375 | A: 0.000 | 0.97 |
| rs1113132 | *EXT2* | C^a^/G | 1.15 (2) | 0.62 | G: 0.421 | G: 0.352 | G: 0.344 | G: 0.300 | G: 0.075 | 0.94 |
| rs11037909 | *EXT2* | T^a^/C | 1.27 (2) | 0.57 | C: 0.422 | C: 0.344 | C: 0.333 | C: 0.300 | C:0.158 | 0.99 |
| rs3740878 | *EXT2* | A^a^/G | 1.26 (2) | 0.02 | G: 0.450 | G: 0.344 | G: 0.341 | G: 0.302 | G: 0.076 | 0.99 |
| rs13266634 | *SLC30A8* | C/T^a^ | 1.22 (13) | 0.01 | T: 0.469 | T: 0.478 | T: 0.444 | T:0.250 | T: 0.058 | 0.99 |
| rs2466293 | *SLC30A8* | A/G^a^ | 1.16 (13) | 0.44 | G: 0.359 | G: 354 | G: 0.389 | G: 0.417 | G: 0.100 | 0.94 |
| Rs7501939 | *TCF2* | C/T^a^ | 1.19 (26) | 0.94 | T: 0.258 | T: 0.284 | T:0.300 | T:0.467 | C:0.458 | 0.96 |
| Rs2237892 | *KCNQ1* | ^a^C/T | 1.40 (23) | 0.34 | T: 0.355 | T:0.367 | T:0.389 | T:0.075 | T:0.100 | 0.99 |
| Rs10830963 | *MTNR1B* | C/G^a^ | 1.09 (24) | 0.03 | G: 0.409 | G: 0.478 | G: 0.500 | G: 0.300 | G:0.05 | 0.56 |
| Rs1387153 | *MTNR1B* | C/T^a^ | 1.15 (25) | 0.48 | T: 0.409 | T: 0.478 | T: 0.522 | T: 0.283 | T:0.425 | 0.93 |

^a^ Reported risk allele in previous studies. The underlined allele is the risk allele in the present study. OR, odds ratio. HWE, Hardy-Weinberg equilibrium. MAF, minor allele frequency. The MAF of CHB, JPT, CEU, YRI was cited from Hap Map project. * Current pooled sample size, minor allele frequencies observed in the present study and the previously reported ORs (Refs) for type 2 diabetes were used for statistical power estimation.
