# Supplementary material for: Combined Effects of 19 Common Variations on Type 2 Diabetes in Chinese: Results from Two Community-Based Studies
Source: PLoS One. 2010 Nov 17;5(11):e14022. doi: 10.1371/journal.pone.0014022 (PMC2984434; doi:10.1371/journal.pone.0014022)
Supplement: Table S2 — The baseline clinical characteristics of the participants in the prospective study. (0.04 MB DOC) [file pone.0014022.s002.doc]

**Table S2.**

|  | Diabetes status at follow-up examination | | |
| --- | --- | --- | --- |
|  | Non-diabetes | Incident Diabetes | *P-*value * |
| Male/Female (M, %) | 245/422 (36.7) | 24/43 (35.8) | 0.88 |
| Age (years) | 61.0 ± 9.0 | 61.5 ± 8.6 | 0.66 |
| Body mass index (kg/m^2^) | 24.8 ± 3.2 | 26.1 ± 3.3 | 0.0008 |
| Systolic blood pressure (mmHg) | 135 ± 24 | 142 ± 24 | 0.03 |
| Diastolic blood pressure (mmHg) | 78 ± 12 | 82 ± 11 | 0.03 |
| Fasting plasma glucose (mmol/l) | 5.4 ± 0.6 | 6.0 ± 0.6 | <0.0001 |
| OGTT-2h plasma glucose (mmol/l) | 6.7 ± 1.5 | 8.5 ± 1.8 | <0.0001 |
| Fasting plasma insulin (μU/ml) | 4.3 (2.2-7.6) | 8.7 (3.4-15.1) | <0.0001 |
| Current smoking, yes, n (%) | 138 (20.7) | 12 (17.9) | 0.75 |
| Current alcohol intake, yes, n (%) | 91 (13.6) | 11 (16.4) | 0.58 |
| Diabetes family history, yes, n (%) | 64 (9.6) | 10 (14.9) | 0.19 |

Data are means ± standard deviation or geometric means (95 %CI). *Based on ANOVA for continuous variables and χ^2^ or fisher exact tests for categorical variables.
