# Supplementary material for: Combined Effects of 19 Common Variations on Type 2 Diabetes in Chinese: Results from Two Community-Based Studies
Source: PLoS One. 2010 Nov 17;5(11):e14022. doi: 10.1371/journal.pone.0014022 (PMC2984434; doi:10.1371/journal.pone.0014022)
Supplement: Table S3 — Standardized pair-wise linkage disequilibrium coefficients D' and r2 of the SNPs. (0.05 MB DOC) [file pone.0014022.s003.doc]

**Table S3.**

| SNP ID | db SNP ID | Chr | Gene | Position | Pair-wise SNPs | D’ | *r^2^* |
| --- | --- | --- | --- | --- | --- | --- | --- |
| SNP 1 | rs1470579 | 3 | *IGF2BP2* | 187011782 | SNP 1 vs. SNP 2 | 0.99 | 0.93 |
| SNP 2 | rs4402960 | 3 | *IGF2BP2* | 186994389 |  |  |  |
| SNP 3 | rs13266634 | 8 | *SLC30A8* | 31402961 | SNP 3 vs. SNP 4 | 0.99 | 0.49 |
| SNP 4 | rs2466293 | 8 | *SLC30A8* | 31404116 |  |  |  |
| SNP 5 | rs10811661 | 9 | *CDKN2A/2B* | 22124094 | SNP 5 vs. SNP 6 | 0.80 | 0.00 |
| SNP 6 | rs564398 | 9 | *CDKN2A/2B* | 22019547 |  |  |  |
| SNP 7 | rs1111875 | 10 | *HHEX* | 94452862 | SNP 7 vs. SNP 8 | 0.97 | 0.51 |
| SNP 8 | rs5015480 | 10 | *HHEX* | 94455539 | SNP 7 vs. SNP 9 | 0.52 | 0.19 |
| SNP 9 | rs7923837 | 10 | *HHEX* | 94471897 | SNP 8 vs. SNP 9 | 0.77 | 0.46 |
| SNP 10 | rs1113132 | 11 | *EXT2* | 44209979 | SNP 10 vs. SNP 11 | 0.98 | 0.97 |
| SNP 11 | rs11037909 | 11 | *EXT2* | 44212190 | SNP 10 vs. SNP 12 | 0.98 | 0.96 |
| SNP 12 | rs3740878 | 11 | *EXT2* | 44214378 | SNP 11 vs. SNP 12 | 0.99 | 0.97 |
| SNP 13 | rs10830963 | 11 | *MTNR1B* | 4970332 | SNP 13 vs. SNP 14 | 0.00 | 0.00 |
| SNP 14 | rs1387153 | 11 | *MTNR1B* | 4935450 |  |  |  |

Chromosome position and allele nomenclature are indicated using NCBI dbSNP build 35. Genotype data of all the participants were used for linkage disequilibrium (LD) estimation (pair-wise D' and *r^2^*).
