# Supplementary material for: Combined Effects of 19 Common Variations on Type 2 Diabetes in Chinese: Results from Two Community-Based Studies
Source: PLoS One. 2010 Nov 17;5(11):e14022. doi: 10.1371/journal.pone.0014022 (PMC2984434; doi:10.1371/journal.pone.0014022)
Supplement: Table S4 — The risk of type 2 diabetes in relation to gene risk scores by stratification analysis. (0.11 MB DOC) [file pone.0014022.s004.doc]

**Table S4**

|  | | | Model 1 | Model 2 | Model 3 |
| --- | --- | --- | --- | --- | --- |
| **Body Mass Index (kg/m^2^)** (*P*_for interaction_ = 0.03) | | | |  |  |
| **≤ 22.9** | (n = 894) |  | |  |  |
|  | Q1 (0-3) | 1.00 | | 1.00 | 1.00 |
|  | Q2 (4) | 1.62 (1.09-2.38) | | 1.67 (1.11-2.52) | 1.65 (1.08-2.53) |
|  | Q3 (5) | 2.17 (1.47-3.20) | | 2.33 (1.55-3.49) | 2.18 (1.43-3.33) |
|  | Q4 (6-8) | 3.02 (1.97-4.61) | | 3.17 (2.03-4.95) | 2.95 (1.85-4.69) |
|  | *P _for trend_* | < 0.0001 | | < 0.0001 | < 0.0001 |
| **23.0-25.0** | (n = 844) |  | |  |  |
|  | Q1 (0-3) | 1.00 | | 1.00 | 1.00 |
|  | Q2 (4) | 1.38 (0.94-2.02) | | 1.32 (0.89-1.96) | 1.39 (0.93-2.10) |
|  | Q3 (5) | 2.35 (1.61-3.43) | | 2.34 (1.58-3.44) | 2.46 (1.63-3.70) |
|  | Q4 (6-8) | 2.86 (1.89-4.37) | | 2.89 (1.87-4.47) | 2.87 (1.82-4.51) |
|  | *P _for trend_* | < 0.0001 | | < 0.0001 | < 0.0001 |
| **25.1-27.4** | (n = 806) |  | |  |  |
|  | Q1 (0-3) | 1.00 | | 1.00 | 1.00 |
|  | Q2 (4) | 1.98 (1.35-2.89) | | 1.93 (1.31-2.84) | 1.95 (1.30-2.91) |
|  | Q3 (5) | 2.73 (0.40-2.87) | | 2.72 (1.82-4.06) | 2.54 (1.68-3.84) |
|  | Q4 (6-8) | 2.31 (1.54-3.45) | | 2.30 (1.53-3.48) | 2.23 (1.46-3.41) |
|  | *P _for trend_* | < 0.0001 | | < 0.0001 | < 0.0001 |
| **≥ 27.5** | (n = 833) |  | |  |  |
|  | Q1 (0-3) | 1.00 | | 1.00 | 1.00 |
|  | Q2 (4) | 1.10 (0.77-1.58) | | 1.08 (0.75-1.57) | 1.13 (0.77-1.64) |
|  | Q3 (5) | 1.87 (1.25-2.79) | | 1.84 (1.22-2.78) | 1.84 (1.21-2.81) |
|  | Q4 (6-8) | 1.45 (0.95-2.23) | | 1.53 (0.99-2.38) | 1.51 (0.97-2.37) |
|  | *P _for trend_* | 0.013 | | < 0.0001 | < 0.0001 |
|  |  |  | |  |  |
| **To be continued** | | | | |  |
| **HOMA_β (%)** (*P*_for interaction_ = 0.35) | | | |  |  |
| ≤ 39.1 | (n = 935) |  | |  |  |
|  | Q1 (0-3) | 1.00 | | 1.00 | 1.00 |
|  | Q2 (4) | 1.32 (0.91-1.92) | | 1.36 (0.87-1.97) | 1.50 (1.23-1.82) |
|  | Q3 (5) | 2.19 (1.51-3.19) | | 2.30 (1.52-3.47) | 2.21 (1.80-2.71) |
|  | Q4 (6-8) | 2.63 (1.74-3.99 | | 2.57 (1.63-4.04) | 2.28 (1.83-2.85) |
|  | *P _for trend_* | < 0.0001 | | < 0.0001 | < 0.0001 |
| 39.2-69.4, | (n = 854) |  | |  |  |
|  | Q1 (0-3) | 1.00 | | 1.00 | 1.00 |
|  | Q2 (4) | 1.23 (0.86-1.76) | | 1.42 (0.94-2.15) | 1.54 (1.00-2.37) |
|  | Q3 (5) | 1.62 (1.12-2.34) | | 1.92 (1.25-2.93) | 1.95 (1.24-3.06) |
|  | Q4 (6-8) | 1.74 (1.15-2.62) | | 2.06 (1.28-3.30) | 2.17 (1.32-3.56) |
|  | *P _for trend_* | 0.02 | | 0.006 | 0.006 |
| 69.5-114.6 | (n = 830) |  | |  |  |
|  | Q1 (0-3) | 1.00 | | 1.00 | 1.00 |
|  | Q2 (4) | 1.81 (1.24-2.63) | | 2.05 (1.33-3.16) | 2.03 (1.29-3.17) |
|  | Q3 (5) | 1.50 (0.99-2.26) | | 1.76 (1.10-2.81) | 1.76 (1.09-2.84) |
|  | Q4 (6-8) | 1.75 (1.14-2.69) | | 1.54 (0.94-2.50) | 1.38 (0.83-2.28) |
|  | *P _for trend_* | 0.009 | | 0.009 | 0.01 |
| ≥ 114.7 | (n = 894) |  | |  |  |
|  | Q1 (0-3) | 1.00 | | 1.00 | 1.00 |
|  | Q2 (4) | 1.30 (0.89-1.91) | | 1.21 (0.80-1.82) | 1.30 (0.86-1.97) |
|  | Q3 (5) | 2.24 (1.51-3.34) | | 2.36 (1.55-3.60) | 2.35 (1.52-3.62) |
|  | Q4 (6-8) | 2.12 (1.35-3.31) | | 2.31 (1.44-3.71) | 2.42 (1.50-3.93) |
|  | *P _for trend_* | < 0.0001 | | < 0.0001 | < 0.0001 |
|  | | | | |  |
| **To be continued** | | | | |  |
| **Diabetes family history** (*P*_for interaction_ = 0.12) | | | | |  |
| **Yes** | (n = 599) |  | |  |  |
|  | Q1 (0-3) | 1.00 | | 1.00 | 1.00 |
|  | Q2 (4) | 2.14 (1.36-3.37) | | 2.55 (1.58-4.12) | 2.57 (1.59-4.17) |
|  | Q3 (5) | 3.11 (1.96-4.94) | | 3.67 (2.26-5.97) | 3.67 (2.25-5.97) |
|  | Q4 (6-8) | 2.94 (1.79-4.84) | | 3.15 (1.87-5.29) | 3.14 (1.87-5.28) |
|  | *P _for trend_* | < 0.0001 | | < 0.0001 | < 0.0001 |
| **No** | (n = 2715) | | |  |  |
|  | Q1 (0-3) | 1.00 | | 1.00 | 1.00 |
|  | Q2 (4) | 1.33 (1.07-1.65) | | 1.33 (1.07-1.65) | 1.37 (1.12-1.68) |
|  | Q3 (5) | 1.98 (1.58-2.48) | | 1.98 (1.58-2.48) | 1.90 (1.54-2.34) |
|  | Q4 (6-8) | 2.12 (1.66-2.72) | | 2.13 (1.66-2.72) | 2.05 (1.63-2.58) |
|  | *P _for trend_* | < 0.0001 | | < 0.0001 | < 0.0001 |

Values are odds ratio (95% confidence interval). *P* *_for trend_* values, for the risk of incident type 2 diabetes, we defined subjects with normal glucose regulation as 0 and type 2 diabetic patients as 1. The study subjects were stratified by quartiles of BMI (≤ 22.9, 23.0-25.0, 25.1-27.4, ≥ 27.5), quartiles of HOMA_β (≤ 39.1, 39.2-69.4, 69.5-114.6, ≥ 114.7) and family history (yes or no). Q1, quartile 1; Q2, quartile 2; Q3, quartile 3; Q4, quartile 4.

Model 1, unadjusted;

Model 2, adjusted for age, gender BMI;

Model.3, adjusted for age, gender BMI, diabetes family history, current smoking and alcohol.
